# Efficacy of texture and color enhancement imaging for the visibility and diagnostic accuracy of non‐polypoid colorectal lesions

**DOI:** 10.1002/deo2.380

**Published:** 2024-05-29

**Authors:** Taishi Okumura, Kinichi Hotta, Kenichiro Imai, Sayo Ito, Yoshihiro Kishida, Kazunori Takada, Daiki Kawaguchi, Yukihiro Mori, Yusuke Tanaka, Takahiro Tsushima, Noboru Kawata, Yuki Maeda, Masao Yoshida, Yoichi Yamamoto, Tatsunori Minamide, Hirotoshi Ishiwatari, Junya Sato, Hiroyuki Matsubayashi, Hiroyuki Ono

**Affiliations:** ^1^ Division of Endoscopy Shizuoka Cancer Center Shizuoka Japan; ^2^ Division of Gastrointestinal Oncology Shizuoka Cancer Center Shizuoka Japan; ^3^ Division of Colon and Rectal Surgery Shizuoka Cancer Center Shizuoka Japan

**Keywords:** colorectal neoplasms, endoscopy, humans, narrow‐band imaging, polyps

## Abstract

**Objective:**

A newly launched endoscopy system (EVIS X1, CV‐1500; Olympus) is equipped with texture and color enhancement imaging (TXI). We aimed to investigate the efficacy of TXI for the visibility and diagnostic accuracy of non‐polypoid colorectal lesions.

**Methods:**

We examined 100 non‐polypoid lesions in 42 patients from the same position, angle, and distance of the view in three modes: white light imaging (WLI), narrow‐band imaging (NBI), and TXI. The primary outcome was to compare polyp visibility in the three modes using subjective polyp visibility score and objective color difference values. The secondary outcome was to compare the diagnostic accuracy without magnification.

**Results:**

Overall, the visibility score of TXI was significantly higher than that of WLI (3.7 ± 1.1 vs. 3.6 ± 1.1; *p* = 0.008) and lower than that of NBI (3.7 ± 1.1 vs. 3.8 ± 1.1; *p* = 0.013). Color difference values of TXI were higher than those of WLI (11.5 ± 6.9 vs. 9.1 ± 5.4; *p* < 0.001) and lower than those of NBI (11.5 ± 6.9 vs. 13.1 ± 7.7; *p* = 0.002). No significant differences in TXI and NBI (visibility score: 3.7 ± 1.0 vs. 3.8 ± 1.1; *p* = 0.833, color difference values: 11.6 ± 7.1 vs. 12.9 ± 8.3; *p* = 0.099) were observed for neoplastic lesions. Moreover, the diagnostic accuracy of TXI was significantly higher than that of NBI (65.5% vs. 57.6%, *p* = 0.012) for neoplastic lesions.

**Conclusions:**

TXI demonstrated higher visibility than that of WLI and lower than that of NBI. Further investigations are warranted to validate the performance of the TXI mode comprehensively.

## INTRODUCTION

Endoscopic resection of adenomatous polyps, which are the precursor lesions of colorectal cancer (CRC), is the most effective measure to prevent CRC.^1^, ^2^ However, according to a recent meta‐analysis including over 15,000 tandem colonoscopies, the miss rates for adenomas, most of which were non‐polypoid lesions, was approximately 26%.^3^ To reduce the incidence of missed lesions in the visual field, the efficacy of image‐enhanced endoscopy (IEE) has been improved using novel techniques, such as narrow‐band imaging (NBI), autofluorescence imaging, and linked color imaging.^4^, ^5^, ^6^ Notably, NBI is reportedly useful in detecting missed lesions^7^; however, it is not the standard for routine colonoscopy withdrawal.

The EVIS X1 (CV‐1500; Olympus Co.), an endoscopic system that uses light‐emitting diodes of five colors as the light source, was released worldwide in 2020. This system has a new observational mode, texture and color enhancement imaging (TXI), which is closer to white light imaging (WLI) than NBI. TXI is a novel IEE technique designed to improve lesion visibility by enhancing three image factors (texture, brightness, and color) to define subtle tissue differences. There are two types of TXI, namely TXI1 and TXI2.^8^, ^9^, ^10^, ^11^ Regarding the basis of the TXI algorithm, the image obtained from WLI is divided into a base image and a texture image. Then, the texture and brightness of these two images are adjusted, and they are combined to create TXI2. Finally, color enhancement is performed to create the TXI1 image.^9^, ^12^

TXI has been used for a relatively short duration of time. Therefore, reports of improved visibility and diagnostic accuracy (DA) for assessing colorectal lesions remain limited. Thus, we aimed to determine whether TXI can improve the visibility of nonpolypoid lesions. In addition, we also examined the DA of TXI without magnification.

### Patients/materials and methods

### Study design

**FIGURE 1:**
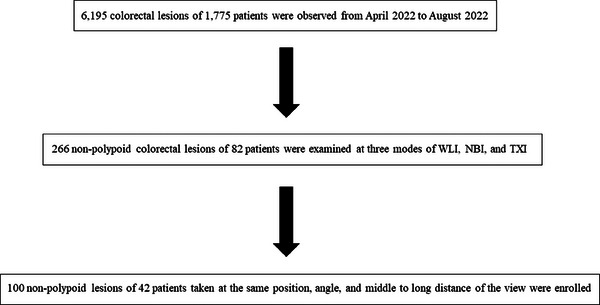
Flowchart depicting the workflow of the study. NBI, narrow‐band imaging; TXI, texture and color enhancement imaging; WLI, white light imaging.

This study was a retrospective, single‐center study. A total of 6195 colorectal lesions were detected in 1775 consecutive patients between April 2022 and August 2022 at the Shizuoka Cancer Center, Shizuoka, Japan. Of these, 266 non‐polypoid colorectal lesions in 82 patients were examined by three modes (WLI, NBI, and TXI) using a dedicated endoscope (CF‐XZ1200I and CF‐EZ1500DI) with the new endoscopic system (EVIS X1). TXI1 was adopted, as it enhances color better than TXI2; furthermore, the visibility in TXI1 is higher than that in TXI2 for colorectal lesions.^11^ A total of 100 non‐polypoid lesions in 42 patients taken from the most identical position, angle, and middle to long distance of the view in the three modes were included in this study (shown in Figure 1). All images were selected by one endoscopist (Taishi Okumura) from those not taken by him.

### Clinicopathologic features

We assessed the clinicopathological features of the patients, including age, sex, location of the lesion, size, morphological type, and histopathological findings. The location of lesions was classified into three categories: right‐sided colon, including the cecum, ascending, and transverse colon; left‐sided colon, including the descending and sigmoid colon; and rectum. The morphological types of the lesions were determined according to the Paris classification.^13^ All lesions were fixed in 10% formalin and histopathologically evaluated. Histopathological diagnoses were performed by several board‐certified pathologists. Diagnostic criteria were based on the Japanese Classification of Colorectal Carcinoma.^14^ Serrated lesions (SLs) were diagnosed based on the latest World Health Organization criteria.^15^

### Outcome measures

A total of 300 images without magnification, representing the three modes of 100 lesions, were randomly and anonymously arranged. All endoscopic images were formatted as Joint Photographic Experts Group images, and ≥20‐inch monitors were used. The primary outcome of this study was to compare the polyp visibility of the three modes using the subjective polyp visibility score (VS) and objective color difference (CD) values. The secondary outcome was the comparison of the DA of the three modes without magnification. Ten endoscopists examined the VS and DA. Of the 10 endoscopists, five (D.K., Y.M., Y.T., T.T., and J.S.) were classified as novices (had performed < 500 colonoscopies and 1‐3 years of experience as gastroenterologists) and had no experience with NBI and TXI. The other five (N.K., Y.M., M.Y., Y.Y., and T.M.) were classified as experts (had performed > 5000 colonoscopies) and had experience with NBI and TXI. The evaluators were not involved in the colonoscopy during the study period and were blinded to the patient background and histological findings.

### Subjective polyp VS

The subjective VS of each image was evaluated using the following criteria. The images were judged on a scale of 1–5 (1: recognition of the lesion was impossible; 2: poor; 3: acceptable; 4: good; and 5: best). The mean VS was compared in the three modes and assessed based on the experience of the endoscopist and histological findings.

### Objective CD

**FIGURE 2:**
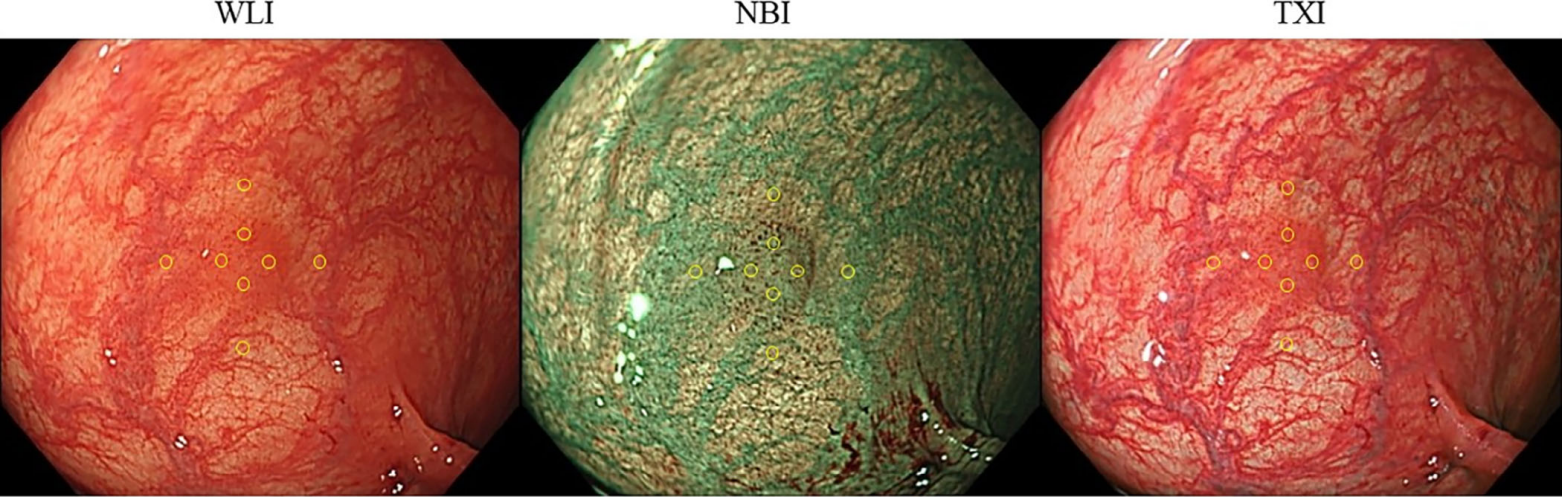
Calculation of CD values of low‐grade adenoma using WLI, NBI, and TXI. The CD value between each polyp and the surrounding mucosa in four directions (north, east, south, and west) was then calculated. CD, color difference; NBI, narrow‐band imaging; TXI, texture and color enhancement imaging; WLI, white light imaging.

The objective CD value of each image was evaluated using the following criteria. CD values can be calculated using the three attributes of color: lightness, chroma, and hue, and they have been positively correlated with increased polyp visibility.^16^, ^17^, ^18^ The color in the images was evaluated using the International Commission on Illumination L*a*b* (CIELAB) color space system,^19^ which is a three‐dimensional space for presenting a color with axes of L* (from black to white; white is highest), a* (from green to red; red is highest), and b* (from blue to yellow; yellow is highest). The distance between two points in the CIELAB color space is proportional to the difference in color perception. The perceived CD of the area between the peripheral and inner points was assessed using the formula: ΔE = [(ΔL*) 2 + (Δa *) 2 + (Δb *) 2] 1/2. ΔE was measured using Photoshop CS5 (version 12.0; Adobe). The region of interest on both the peripheral and inner points was defined as follows (shown in Figure 2). Initially, the margins and center of a polyp were identified. The four equal peripheral non‐polyp points 3 mm outside the lesion (north, east, south, and west) were identified. The four inner points were annotated at two‐thirds of the distance from the peripheral points to the center of the lesion. CD values were calculated for each of the four ΔE (the peripheral and inner points). The mean CD values of the four areas per lesion (mean ΔE) were analyzed. The CD values of the three modes were examined according to the histopathological characteristics of the lesion.

### Diagnostic accuracy

None of the images were magnified, and the DA was evaluated. The evaluators immediately answered the question with their first impressions based on the morphology and texture of the lesion in four categories: hyperplastic polyp or sessile serrated lesion, adenoma, Tis (intramucosal cancer) or T1a (submucosal invasive cancer <1000 εm), and T1b (submucosal invasive cancer ≥1000 εm).^14^, ^15^ Mean DA was compared in three modes and assessed according to the experience of the endoscopist and histological findings.

### Statement of Ethics

This study was conducted in accordance with the tenets of the Declaration of Helsinki. The study protocol was approved by the Institutional Review Board of Shizuoka Cancer Center (J‐2022‐128‐2022‐1‐3). The opt‐out method was used to obtain consent from the study participants.

### Statistical analysis

All statistical analyses were performed using EZR (version 1.56; Saitama Medical Center, Jichi Medical University), a graphical user interface for R software (The R Foundation for Statistical Computing). To determine differences between the three groups (WLI vs. NBI vs. TXI), continuous and categorical variables were compared using one‐way analysis of variance and Fisher's exact tests, respectively, followed by multiple comparison testing using the Bonferroni–Dunn method. *p* < 0.05 was considered statistically significant.

## RESULTS

### Clinicopathological characteristics

The clinicopathological characteristics of the enrolled patients and their lesions are presented in Table 1. The mean (standard deviation) age of the patients was 65.9 (± 10.5) years, and the male‐to‐female ratio was 34:8. Seventy‐nine lesions (79.0%) measured <10 mm. Ninety‐five 0‐IIa lesions (95.0%) were included in the analysis. Sixty‐five lesions (65.0%) were located in the right colon. We observed 12 hyperplastic polyps, 22 sessile SLs (SSLs), 56 low‐grade adenomas, four high‐grade adenomas, three Tis tumors, and three T1 tumors.

**TABLE 1:** Clinicopathologic characteristics of enrolled patients and lesions.

Variables	Total (%)
**Patients, *n***	42
**Age, mean ± SD**	65.9 ± 10.5
**Sex**	
Male	34 (81.0)
Female	8 (19.0)
**Lesions, *n***	100
**Polyp size**	
<10 mm	79 (79.0)
≥10 mm	21 (21.0)
**Morphology**	
IIa	95 (95.0)
IIa+IIc	2 (2.0)
IIc	3 (3.0)
**Location**	
Right‐sided colon	65 (65.0)
Left‐sided colon	33 (33.0)
Rectum	2 (2.0)
**Histopathological diagnosis**	
Hyperplastic polyp	12 (12.0)
Sessile serrated lesion	22 (22.0)
Low‐grade adenoma	56 (56.0)
High‐grade adenoma	4 (4.0)
Tis	3 (3.0)
T1	3 (3.0)

Abbreviations: SD, standard deviation; T1, submucosal invasive cancer; Tis, intramucosal cancer.

### Polyp visibility

**FIGURE 3:**
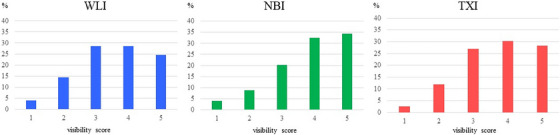
The percentage of subjective VS responses to each category when all endoscopists evaluated all lesions. The images were judged on a scale of 1–5 (1: recognition of the lesion was impossible; 2: poor; 3: acceptable; 4: good; and 5: best). NBI, narrow‐band imaging; TXI, texture and color enhancement imaging; VS, visibility score; WLI, white light imaging.

The VS values of the three modes are listed in Table 2. Overall, the VS of TXI was significantly higher than that of WLI (3.7 ± 1.1 vs. 3.6 ± 1.1; *p* = 0.008) and lower than that of NBI (3.7 ± 1.1 vs. 3.8 ± 1.1; *p* = 0.013). The percentage of subjective VS responses to each category when all endoscopists evaluated all lesions is shown in Figure 3. In only NBI, the highest percentage of respondents judged on a scale of 5. For novices, no significant difference was observed between TXI and WLI (3.4 ± 1.2 vs. 3.4 ± 1.2; *p* = 1.0). For experts, the VS of TXI was significantly higher than that of WLI (4.0 ± 0.9 vs. 3.7 ± 1.0; *p* < 0.001). There was no significant difference in TXI and NBI (3.7 ± 1.1 vs. 3.9 ± 1.1; *p* = 0.095) for SLs. There was no significant difference in TXI or NBI (3.7 ± 1.0 vs. 3.8 ± 1.1; *p* = 0.833) for neoplastic lesions.

**TABLE 2:** Polyp visibility score of white light imaging (WLI), texture and color enhancement imaging (TXI), and narrow‐band imaging (NBI) by endoscopists.

Variables	WLI	TXI	NBI	*p‐*value WLI versus TXI	*p‐*value TXI versus NBI	*p‐*value WLI versus NBI
Overall	3.6 ± 1.1	3.7 ± 1.1	3.8 ± 1.1	0.008	0.013	<0.001
Polyp size <10 mm	3.5 ± 1.1	3.7 ± 1.1	3.8 ± 1.1	0.001	0.023	<0.001
Polyp size ≥10 mm	3.8 ± 1.1	4.0 ± 1.0	4.1 ± 1.1	0.046	0.712	0.020
Novice	3.4 ± 1.2	3.4 ± 1.2	3.6 ± 1.2	1.00	0.11	0.05
Expert	3.7 ± 1.0	4.0 ± 0.9	4.1 ± 0.9	<0.001	0.12	<0.001
HP+SSL	3.5 ± 1.2	3.7 ± 1.1	3.9 ± 1.1	0.095	0.095	<0.001
LGA + HGA + Tis + T1	3.6 ± 1.1	3.7 ± 1.0	3.8 ± 1.1	0.007	0.833	<0.001

Abbreviations: HGA, high‐grade adenoma; HP, hyperplastic polyp; LGA, low‐grade adenoma; NBI, narrow‐band imaging; SSL, sessile serrated lesion; TXI, texture and color enhancement imaging; WLI, white light imaging.

The CD values of the three modes are listed in Table 3. Overall, the CD values of TXI were higher than those of WLI (11.5 ± 6.9 vs. 9.1 ± 5.4; *p* < 0.001) and lower than those of NBI (11.5 ± 6.9 vs. 13.1 ± 7.7; *p* = 0.002). For SLs, no significant difference was observed between TXI and WLI (11.2 ± 6.5 vs. 9.9 ± 5.7; *p* = 0.262). Furthermore, TXI was significantly lower than NBI (11.2 ± 6.5 vs. 13.5 ± 6.5; *p* = 0.01) for SLs. For neoplastic lesions, we observed no significant difference between TXI and NBI (11.6 ± 7.1 vs. 12.9 ± 8.3; *p* = 0.099).

**TABLE 3:** Color difference values of the three modes according to histopathological lesion characteristics.

Variables	WLI	TXI	NBI	*p‐*value WLI versus TXI	*p‐*value TXI versus NBI	*p‐*Value WLI versus NBI
All	9.1 ± 5.4	11.5 ± 6.9	13.1 ± 7.7	<0.001	0.002	<0.001
HP + vSSL	9.9 ± 5.7	11.2 ± 6.5	13.5 ± 6.5	0.262	0.01	<0.001
LGA + HGA + Tis + T1	8.7 ± 5.1	11.6 ± 7.1	12.9 ± 8.3	<0.001	0.099	<0.001

Abbreviations: HGA, high‐grade adenoma; HP, hyperplastic polyp; LGA, low‐grade adenoma; NBI, narrow‐band imaging; SSL, sessile serrated lesion; TXI, texture and color enhancement imaging; WLI, white light imaging.

### Diagnostic accuracy

**FIGURE 4:**
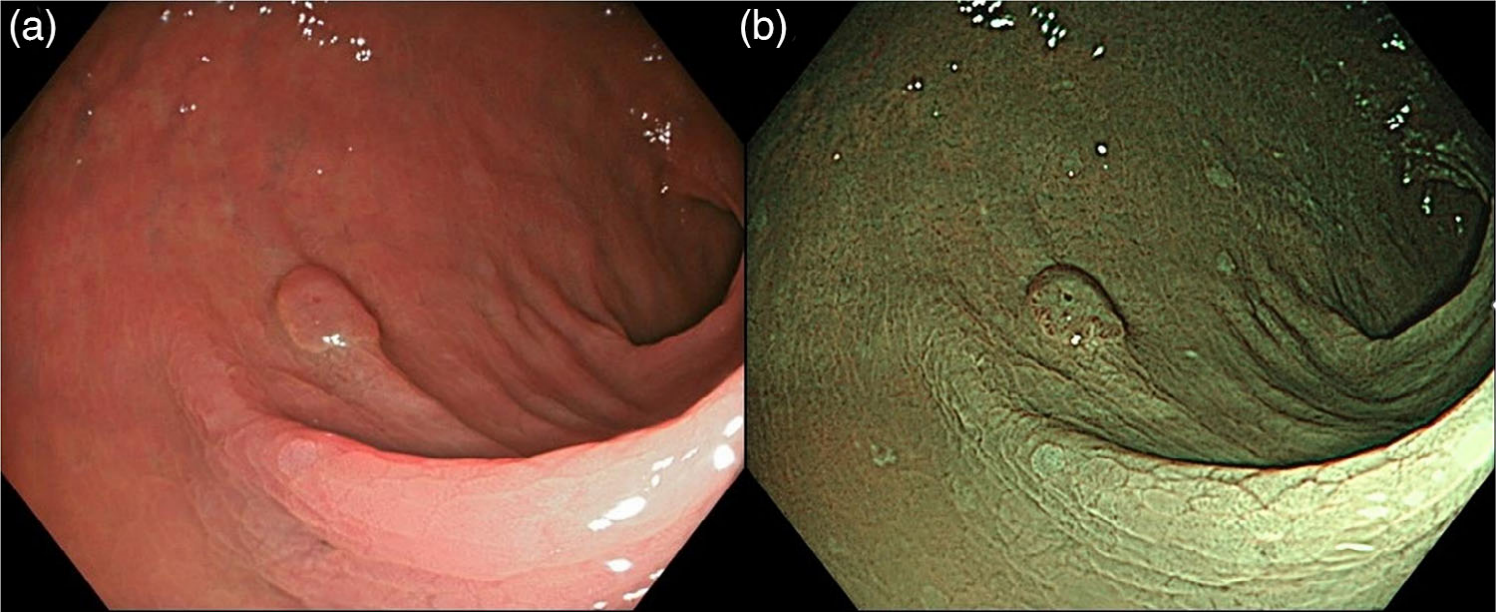
Representative case lacking changes in the VS and the CD values but with improvement in the DA of TXI. (a) TXI revealed that the mean DA, VS, and ΔE were 70%, 4.0, and 11.6, respectively. (b) NBI indicated that the mean DA, VS, and ΔE were 40%, 4.1, and 11.5, respectively. CD, color difference; DA, diagnostic accuracy; NBI, narrow‐band imaging; TXI, texture and color enhancement imaging; VS, visibility score.

The DA values of the three modes are listed in Table 4. Overall, DA revealed no significant difference among the three modes (60.8% in TXI vs. 57.0% in NBI vs. 60.8% in WLI). The DA of TXI was significantly higher than that of NBI (50.4% vs. 41.6%; *p* = 0.019) for novices. For experts, the DA indicated no significant difference among the three modes (71.2% for TXI vs. 72.4% for NBI vs. 73.2% for WLI). For SLs, DA did not significantly differ among the three modes (51.8% for TXI vs. 55.9% for NBI vs. 57.1% for WLI). For neoplastic lesions, the DA of TXI was significantly higher than that of NBI (65.5% vs. 57.6%, *p* = 0.012; shown in Figure 4).

**TABLE 4:** Diagnostic accuracy of the three modes by endoscopists.

Variables	WLI	TXI	NBI	*p‐*value WLI versus TXI	*p‐*Value TXI versus NBI	*p‐*value WLI versus NBI
**Overall**	60.8%	60.8%	57.0%	1	0.28	0.28
**Novice**	48.4%	50.4%	41.6%	1	**0.019**	0.108
**Expert**	73.2%	71.2%	72.4%	1	1	1
**HP+SSL**	57.1%	51.8%	55.9%	0.57	0.95	1
**LGA + HGA + Tis + T1**	62.7%	65.5%	57.6%	0.988	**0.012**	0.191

Abbreviations: HGA, high‐grade adenoma; HP, hyperplastic polyp; LGA, low‐grade adenoma; NBI, narrow‐band imaging; SSL, sessile serrated lesion; TXI, texture and color enhancement imaging; WLI, white light imaging.

## DISCUSSION

Our findings demonstrated that the subjective VS and objective CD values of TXI were significantly higher than those of WLI and lower than those of NBI. For neoplastic lesions, the VS or the CD values did not significantly differ between TXI and NBI. For neoplastic lesions, the DA of TXI was significantly higher than that of NBI for all endoscopists. To the best of our knowledge, this is the first study to investigate the efficacy of TXI regarding the visibility and DA of non‐polypoid colorectal neoplastic lesions compared to WLI and NBI.

TXI is a novel IEE designed to improve lesion visibility by enhancing three imaging factors (texture, brightness, and color). Tamai et al.^11^ reported that TXI improved the visualization of colorectal lesions compared with that of WLI using a visual analog scale (0–100). Polyp VS is a subjective indicator. We investigated the indicators of both VS and objective CD values. TXI images had a significantly better overall polyp VS compared to WLI images. Analysis of CD values also revealed similar results, which is because TXI selectively enhances the brightness in the dark areas of an endoscopic image, thereby facilitating the easy identification of lesions that are distant from the endoscope.^8^, ^9^, ^10^, ^11^

Several studies have reported improved visibility with TXI compared to NBI in colorectal lesions.^11^, ^20^ Yoshida et al.^18^ reported the lack of a significant difference in visibility between TXI and NBI in a study that included CD values. However, our findings suggested that NBI was the best of the three modes for mean VS and CD values (3.8 ± 1.1 and 13.1 ± 7.7, respectively). These differences in the results may be attributed to the strengths of this study. Our study involved 10 endoscopists (five novices and five experts), which was a higher number compared with that in previous studies on TXI visibility (four,^11^ six,^18^ and three^20^ endoscopists). We expected to get a fair evaluation by including novices who had little experience with IEE, rather than non‐experts (who had performed 500–5000 colonoscopies). Furthermore, we previously compared the CD values of NBI between EVIS X1 and second‐generation EVIS LUCERA and demonstrated that these values in the NBI‐X1 system were significantly higher than those observed with LUCERA.^21^ Therefore, the novel findings of the current study could be obtained owing to the bright, high‐resolution images of NBI with EVIS X1.

Recently, SSLs have been considered as early precursor lesions in the serrated neoplasia pathway, reportedly accounting for 15%–30% of CRCs.^22^ Nishizawa et al.^20^ reported that TXI provided higher VSs without magnification for SLs, including SSLs than that of WLI and NBI. Our findings also revealed that, although TXI had better VS and CD values in SLs than WLI, there was no significant difference. The small number of SLs (34 lesions) may have affected these results. In this study, in contrast to previous reports, NBI exhibited the most favorable outcomes for SLs, as evidenced by both mean VS and CD values (3.9 ± 1.1 and 13.5 ± 6.5, respectively). Although SLs are subtle polyps with slightly elevated morphology and color similar to that of the surrounding mucosa on endoscopic images, NBI with EVIS X1 can potentially detect such subtle differences. However, the detection rate of proximal SLs using NBI was not significantly higher than that of WLI.^23^ Further additional research is needed on the detection of SSLs.

Our findings indicated that NBI was the worst of the three modes in terms of DA without magnification. Magnified NBI helps comprehensively visualize the blood vessels and capture the surface pattern, resulting in a high positive diagnosis rate.^24^, ^25^ The DA of NBI magnification was reported to be significantly superior to that of TXI magnification (87.1% vs. 80.5%; *p* = 0.027).^18^ Meanwhile, in the present study, the DA of TXI was significantly higher than that of NBI for neoplastic lesions (65.5% vs. 57.6%; *p* = 0.012). The color enhancement algorithm of TXI was designed to better distinguish between red and white hues in the image.^8^ We speculate that the red color of neoplastic blood vessels in middle‐to‐long distance view of non‐magnified TXI provides enhanced contrast compared to the brown color of those in non‐magnified NBI. It may be beneficial to use NBI for lesion detection, switch modes, and characterize the lesions with TXI.

With the development of various IEEs, the future standard mode of withdrawal has become an important topic. The first assumption is that the ADR of IEE must be superior to that of WLI. Recently, a multicenter randomized controlled trial indicated that, compared to WLI, TXI improves the detection of colorectal neoplastic lesions.^26^ This study found that TXI increased the detection of polyps <10 mm in size, both in the proximal and in the distal colon and both in non‐polypoid and polypoid lesions. However, the previous generation of colonoscopes (190 and 290 series) was also included. Our study used only new colonoscopes (CF‐XZ1200I and CF‐EZ1500DI) and showed that NBI has the best visibility. NBI was developed in 2006 and has been preferred for its efficient magnification; however, it has not been widely used for detecting polypoid lesions owing to unfamiliarity and fatigue during withdrawal. Although its efficacy was limited to good bowel preparation, a meta‐analysis revealed that second‐generation NBI had a higher adenoma detection rate than WLI.^7^ To validate the further applicability of TXI in detecting polyps, the ADR should be compared to that of NBI with EVIS X1. TXI may be considered the standard mode in EVIS X1 if its ADR is significantly superior to WLI and equivalent to NBI.

This study had a few limitations. First, this was a single‐center study with a small sample size. Second, although images selected in the three modes were captured from the most identical positions, angles, and distances, a selection bias could have influenced the results. Third, the observation mode of still images was not blinded for evaluators. We used still images rather than videos to examine polyp visibility. Fourth, the improved polyp visibility of TXI may not necessarily lead to improved polyp detection in a clinical setting, as polyp detection is influenced by several factors, in addition to polyp visibility. However, our basic study with three endpoints of VS, CD, and DA values, including a comparison with NBI, has critical implications for improved adenoma detection.

## CONCLUSION

In conclusion, our findings suggested that TXI provided higher visibility than WLI and lower visibility than NBI in terms of the VS and CD values obtained by the endoscopist. The current study is just a pilot study. Therefore, while our study provides novel insights into its efficacy and superiority, further investigations are warranted to validate the performance of the TXI mode comprehensively.

## CONFLICT OF INTEREST STATEMENT

None.

## Data Availability

All data generated or analyzed during this study are included in this article. Further inquiries can be directed to the corresponding author.
